# Evidence of the Existence of Site-Specific Female Contact Pheromones Involved in the Sexual Interaction Behavior of the Pacific Whiteleg Shrimp *Penaeus vannamei*

**DOI:** 10.3390/ani14111523

**Published:** 2024-05-22

**Authors:** José A. Gutiérrez-Vera, Elizabeth Ponce-Rivas, André Braga, Carmen G. Paniagua-Chávez, Jorge Alfaro-Montoya, Misael Rosales-Leija

**Affiliations:** 1 Centro de Investigación Científica y de Educación Superior de Ensenada, Baja California (CICESE), Ensenada 22860, Mexico; jose.gutve@gmail.com (J.A.G.-V.); eponce@cicese.mx (E.P.-R.); cpaniagu@cicese.mx (C.G.P.-C.); 2 Instituto de Investigaciones Oceanológicas, Universidad Autónoma de Baja California, Ensenada 22873, Mexico; andre.braga@uabc.edu.mx; 3 Estación de Biología Marina, Escuela de Ciencias Biológicas, Universidad Nacional, Puntarenas 86-3000, Costa Rica; jalfarom05@gmail.com

**Keywords:** female, contact pheromone, reproduction, shrimp

## Abstract

**Simple Summary:**

If the existence of female sex pheromones in shrimp, *Penaeus vannamei*, is confirmed, they could be used to increase the larval production needed to supply shrimp farms worldwide. To gather more evidence of their existence, liposoluble compounds were extracted from the cuticle of different portions of the abdomen of adult females. These extracts were used to coat PVC tubes that were used as artificial females placed in tanks stocked with adult males. The males were monitored to determine if sexual behavior was displayed when in contact with the artificial females. The behavior observed in the shrimp males of this study provides evidence of the existence of sex pheromones located primarily in the ventral portion of the abdomen exoskeleton of shrimp females.

**Abstract:**

Although the presence of female contact sex pheromones in *P*. *vannamei* has been hypothesized, to date its existence has not been proven. To gather more evidence of their existence, cuticular liposoluble extracts were obtained from the following samples of adult females to be used as the experimental treatments: (1) ventral exoskeleton of immature female (VI), (2) dorsolateral exoskeleton of immature female (DI), (3) ventral exoskeleton of mature female (VM), and (4) dorsolateral exoskeleton of mature female (DM). Polyvinyl chloride tubes (artificial females; AF) were coated with each extract and the behavior displayed by sexually mature males in contact with the AF was recorded and classified as follows: 0 = no response; 1 = contact; 2 = pushing; and 3 = prolonged contact (≥10 s). To test the hypothesis that the extracts collected from the ventral portion of the abdomen exoskeleton have a higher effect on the behavior of males than the extracts collected from the dorsolateral portion of the abdomen exoskeleton, the experiment was divided into two bioassays: Bioassay I (VI vs. DI) and Bioassay II (VM vs. DM). In each bioassay, all experimental treatments were significantly different (*p* > 0.05) from the CTL group (AF coated with hexane). Notably, the pushing behavior was significantly higher (*p* < 0.05) in the VI treatment compared to the CTL and DI treatment. These results provide evidence of the existence of contact female sex pheromones with sexual recognition function located primarily in the ventral portion of the abdomen exoskeleton of *P*. *vannamei*.

## 1. Introduction

The Pacific white shrimp, *Penaeus vannamei*, is the most cultivated crustacean in the world [1], so more research related to the control of reproduction is required to optimize its culture and thus satisfy the need for better quality postlarvae [2]. Among the technologies whose development could contribute to meeting this objective, the use of pheromones emerges as one with great potential, since they are directly involved in various reproductive processes.

A pheromone is defined as a substance released by an individual into the surrounding environment and picked up by other members of the same species, eliciting a specific response [3]. Specifically, sex pheromones are very important in reproduction because they act as a stimulus to initiate or facilitate mating by males or females [4]. Two types of sex pheromones have been identified in crustaceans: proximity (contact) pheromones and distance pheromones [5]. Distance pheromones are soluble in water, act at low concentrations, and are perceived by olfaction. In contrast, contact pheromones are insoluble in water and act in chemoreception at high concentrations [5]. In aquatic crustaceans, contact pheromones are liposoluble compounds, such as waxes and hydrocarbons [4].

Although there has been previous research on crustacean pheromones [4,6,7], these studies have paid little attention to the potential applications of these molecules, covering only preliminary attempts to incorporate pheromones as feed attractants or their use to facilitate the capture of wild broodstock [8,9]; however, the use of pheromones in aquaculture and fisheries has great potential [10,11,12]. Pheromones have the potential to solve several current problems in the captive breeding of crustaceans, either by substituting current techniques or by complementing them to increase their efficiency. To reach this point, however, it is first necessary to confirm the existence of crustacean pheromones and their location to be used as a new resource to improve reproduction.

Dunham (1988) established the necessary conditions for the correct development of behavioral bioassays for the detection of female sex pheromones in crustaceans [13]. These conditions include: (1) limiting the influence of other signaling modalities (sound and visual), (2) the use of negative controls, (3) the use of “blind” assays in which the observer is unaware of the nature of the substances under investigation, and (4) the visualization of a clear behavior. Bamber & Naylor (1996) also established the need to use sexually mature males receptive to female pheromones [14].

Although in *P*. *vannamei*, the identification of sex pheromones has not yet been carried out [2], the existence of sex pheromones in this species has been hypothesized by Misamore and Browdy (1996), when the authors attributed the chasing and mating behaviors displayed by males to the effect of sexual pheromones [15]. Additionally, Yano et al. (1988) hypothesized that chasing and mating behaviors were triggered by pheromones [16]. These authors proposed that the chasing behavior displayed by *P*. *vannamei* was triggered by a distance pheromone that they termed “chase-stimulating pheromone (CSP)”. In contrast, mating behavior was promoted by a contact pheromone that was detected by the male antennules, which they termed “mating-stimulating pheromone (MSP)”. Therefore, studies on these molecules in *P. vannamei* are limited, so many aspects still need to be covered for their application in the aquaculture industry. In the specific case of MSPs, as they are mainly related to mating, a better knowledge of their effect would have an application in the shrimp industry since they could increase the copulation rate of breeding organisms, thus increasing the efficiency of reproduction in captivity.

Thus, the present study aimed to examine the hypothesis that the effect on the mating behavior of male shrimp would be more significant in the presence of an artificial female (AF) coated with extracts collected from the ventral portion of the abdomen exoskeleton. To this end, a series of bioassays were developed to observe and classify the behavior displayed by sexually mature males in contact with AF previously treated with liposoluble cuticular extracts collected from the ventral and dorsolateral portions of the abdomen exoskeleton of sexually mature and immature females.

## 2. Materials and Methods

All methods described next were performed at the Department of Aquaculture of the Centro de Investigación Científica y de Educación Superior de Ensenada, Baja California (CICESE).

### 2.1. Extraction of Liposoluble Cuticular Compounds

Ten adult females (35 ± 6 g) stocked in a maturation tank with natural photoperiod and fed with frozen polychaetes and squid, as well as with a semi-moist formulated feed (Redi-Mate, Zeigler Bros. Inc., Gardners, PA, USA), were monitored daily to determine the gonadal maturation stage according to the observation of the size and pigmentation of the gonad against the light [17]. Females on stage IV of gonadal maturity were removed from their tanks between 12:00 h and 19:00 h to be later sacrificed by cold shock following the procedure approved by the ethical committee of CICESE (ORGA_AQUA_2023_01). It should be noted that the maturation of the females occurred without eyestalk ablation.

The abdomen exoskeleton was collected from females in stage I (immature) and stage IV (mature) of gonadal maturation to evaluate the effect of liposoluble cuticular compounds from immature and mature females on male reproductive behavior. Shrimp sampling for immature females was performed once the females had spawned and finished the gonadal reabsorption to be sure that the females were in stage I of gonadal maturation.

To obtain the cuticular liposoluble extract from the abdomen exoskeleton, the methodology of Zhang et al. (2011) was followed [4], with some modifications described next. First, to test the hypothesis that the extracts collected from the ventral portion of the abdomen exoskeleton have the greatest effect on the mating behavior of male shrimp, each structural component of the ventral and dorsolateral region of the abdomen exoskeleton of each female was equally and carefully collected and washed thoroughly with distilled water. Later, they were macerated separately for a subsequent immersion in 5 mL of HPLC-grade hexane for 30 s. Then, each extract (abdominal and dorsolateral) was passed through a 0.5 mm mesh sieve and subsequently collected in 60 mL amber bottles for storage at −20 °C. This procedure was repeated with ten female shrimp to collect 50 mL of each extract (ventral and dorsolateral) The extracts were stored at 4 °C.

To concentrate the liposoluble extracts and thus increase the probability of response by the males, the solvent from the 50-mL sample was removed completely from each extract by rotary evaporation before the bioassays. The residue was then resuspended in 20 mL of hexane. Once concentrated, each extract was used separately to coat 0.5-inch PVC tubes 9 cm in length, which were later used as artificial females (AF) in the bioassays.

At the end, four different extracts were obtained, corresponding to the experimental treatments: (1) ventral exoskeleton of mature female (VM); (2) dorsolateral exoskeleton of mature female (DM); (3) ventral exoskeleton of immature female (VI); and (4) dorsolateral exoskeleton of immature female (DI). A solution of hexane with no cuticular extract was also used to coat additional AF to be used as the negative control group (CTL).

### 2.2. Bioassays

The experiments were carried out in a recirculating aquaculture system (RAS) filled with filtered seawater (35 ppt). The RAS consisted of 10 fiberglass tanks with a capacity of 150 L each, all connected to a Bubble Bead^®^ filter and a 100 L reservoir tank. Four 300 W temperature controllers were placed in the reservoir tank to maintain a constant temperature of 28 °C. Each tank had one air stone to maintain the dissolved oxygen levels within optimum values for shrimp broodstock. All the bioassays were carried out during the natural photoperiod between 18:00 and 20:00 h.

One haphazardly selected male shrimp was placed into each tank and no individual shrimp was used more than once to avoid habituation. After stocking, a 1-h conditioning period was maintained by placing a plastic mat on top of each tank. For each bioassay, once the conditioning period was over, two AF were consistently submerged into the bottom of each tank approximately 20 inches apart from each other. The preparation of the tubes consisted of immersing them for 1 s in the corresponding treatment, with subsequent drying of the tube for 15 s at ambient temperature (23 °C) [4].

The experimental period of each bioassay was 20 min. During this period, the behavior of the shrimp in the presence of the AF was recorded with a high-resolution quad cellular camera (40 megapixels (MP) + 20 MP + 8 MP + Time of Flight) at 2160 progressive scan resolution and 30 Frames Per Second located on top of the experimental tanks to allow for a clear view of the behavior of males. A blinded observer registered the behaviors displayed by the male shrimp.

To determine the total bioactivity displayed, a behavioral scale from “0” to “4” was generated for the mating behavior stages observed in this study. Table 1 provides a brief description of each behavior stage and the degree within the mating behavioral scale assigned to each stage.

#### 2.2.1. Bioassay I: Effect of Immature Female Extracts on the Mating Behavior of a Male Shrimp

To evaluate the effect of immature female extracts on the behavior of a male shrimp, a bioassay was conducted using 15 replicates for each of the following treatments: AF coated with VI; AF coated with DI; and AF coated with hexane as CTL.

#### 2.2.2. Bioassay II: Effect of Mature Female Extracts on the Mating Behavior of a Male Shrimp

The effect of mature female extracts on the behavior of a male shrimp was evaluated in a bioassay in which 15 replicates were used for each of the following treatments: AF coated with VM; AF coated with DM; and AF coated with hexane as CTL.

### 2.3. Data Collection and Statistical Analysis

The presence or absence of each behavior stage was registered in each replicate, and the number of times each behavior was observed was recorded. Finally, the number of different behavior stages recorded was evaluated as total bioactivity by treatment.

To determine the relationship between each behavior stage and the different treatments, a contingency table analysis (3 × 2) was performed with the Chi-squared (X^2^) test of independence. If the variables were dependent (*p* < 0.05), the Cramer’s V coefficient was calculated. Additionally, for behavior stages that occurred more than once per replicate, the number of times each behavior stage occurred per treatment was analyzed with a Kruskal–Wallis (K–W) test. If the K–W test showed statistically significant results (*p* < 0.05), a pairwise comparison using Dunn’s test with Bonferroni’s adjustment was performed. Finally, total bioactivity data were analyzed by the Kruskal–Wallis test. All statistical analyses were performed using R Studio (v 2023.09.1+494).

## 3. Results

### 3.1. Bioassay I: Effect of Immature Female Extracts on the Mating Behavior of Shrimp Males

When exposed to cuticular extracts from immature females and after initial recognition with the antennal flagellum, *P*. *vannamei* males showed three behavior stages: (1) contact, (2) pushing, or (3) prolonged contact. Supplementary Videos S1 and S2 show a male shrimp displaying the contact and pushing behaviors, respectively, observed in this study.

**Figure 1:**
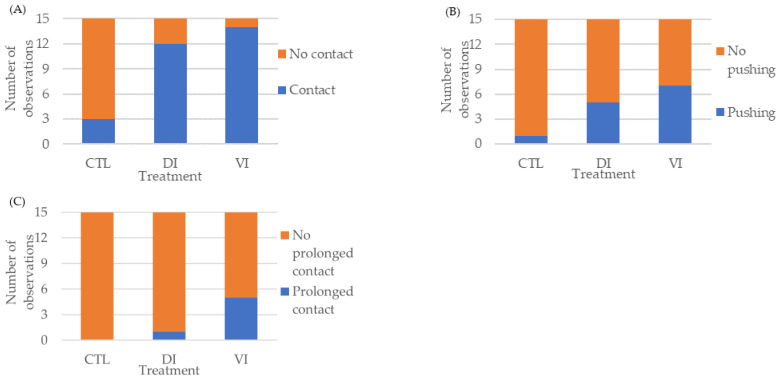
Behavioral response of *Penaeus vannamei* males exposed to artificial females coated with hexane (CTL) or the extracts collected from the different sections of the abdomen exoskeleton of immature females of Bioassay I (*n* = 15 replicates/treatment). Contact (**A**). Pushing (**B**). Prolonged contact (**C**). Abbreviations: CTL, control; DI, dorsolateral exoskeleton collected from the abdomen portion of immature female; VI, ventral exoskeleton collected from the abdomen portion of immature female.

Contact behavior (Figure 1A) was displayed in a significantly higher number of replicates of the DI and VI treatments (12 and 14 replicates, respectively) as compared to the control group (3 replicates) (*p* < 0.01). Additionally, a strong relationship between treatments and contact behavior was detected (Cramer’s V = 0.67). It should be noted that shrimp from the CTL group avoided contact with the AF after their perception with their antennal flagellum; this was not observed in the other two treatments. Supplementary Video S3 shows a male shrimp displaying the avoidance behavior observed only in shrimp from the CTL group.

Pushing behavior (Figure 1B) was also displayed in a significantly higher number of replicates of the DI and VI treatments (5 and 7, respectively), as compared to the control group (1 replicate) (*p* < 0.05). However, a moderate relationship between treatments and the pushing behavior stage was detected (Cramer’s V = 0.37).

Finally, prolonged contact behavior (Figure 1C) was displayed in a significantly higher number of replicates of the VI treatment (5 replicates), as compared to the DI treatment (1 replicate) and the control group (no replicates) (*p* < 0.01). Additionally, a relatively strong relationship between treatments and the prolonged contact behavior stage was detected (Cramer´s V = 0.42).

Among the behavior stages displayed, only contact and pushing occurred more than once in one or more replicates. In the case of the contact behavior stage, there were significant differences (*p* < 0.01) in the number of times this stage was displayed between the CTL and the experimental treatments, but not between each of the experimental treatments (Table 2). On the other hand, the pushing behavior stage was observed significantly more times (*p* < 0.05) in the VI treatment than in the CTL group, with no significant differences between the CTL group and DI treatment or between the DI and VI treatments (Table 2).

**Figure 2:**
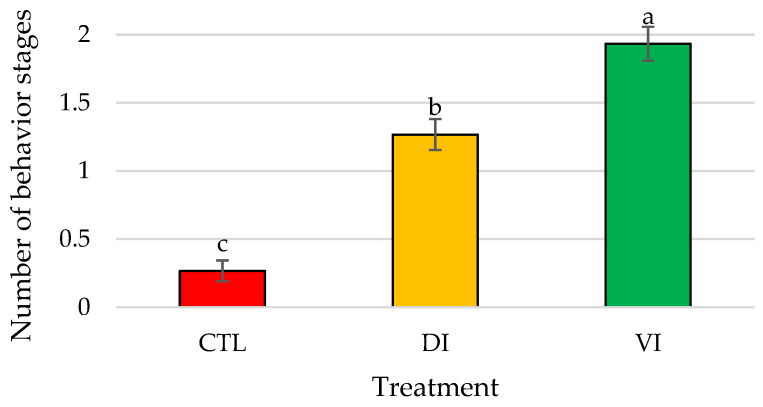
Mean number of different behavior stages recorded expressed as total bioactivity displayed by *Penaeus vannamei* males when exposed to artificial females of Bioassay I (*n* = 15 replicates/treatment); bars represent the standard error of the mean. Different letters indicate significant differences in treatment means (*p* < 0.05). Abbreviations: CTL, control; DI, dorsolateral exoskeleton collected from the abdomen portion of immature female; VI, ventral exoskeleton collected from the abdomen portion of immature female.

Significant differences (*p* < 0.01) were found in the total bioactivity displayed by *P. vannamei* males in the presence of immature female extracts between the three treatments (Figure 2).

### 3.2. Bioassay II: Effect of Mature Female Extracts on the Mating Behavior of Shrimp Males

The males showed only two behavior stages when exposed to cuticular extracts from mature females: (1) contact and (2) pushing. Supplementary Videos S1 and S2 show a male shrimp displaying the contact and pushing behaviors, respectively, observed in this study.

**Figure 3:**
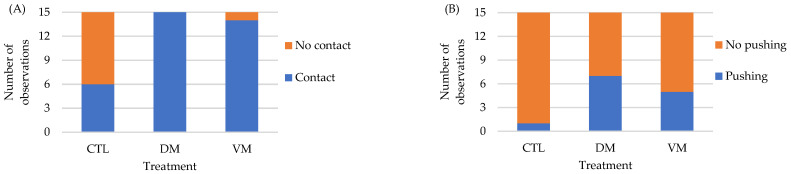
Behavioral response of *Penaeus vannamei* males when exposed to artificial females coated with hexane (CTL) or the extracts collected from the different sections of the abdomen exoskeleton of mature females of Bioassay II (*n* = 15 replicates/treatment). Contact (**A**). Pushing (**B**). Abbreviations: CTL, control; DM, dorsolateral exoskeleton collected from the abdomen portion of mature female; VM, ventral exoskeleton collected from the abdomen portion of mature female.

Contact behavior was displayed in a greater number of replicates of DM and VM treatments (15 and 14 replicates, respectively), as compared to the CTL group (6 replicates) (*p* < 0.01) (Figure 3A). Additionally, a strong relationship between treatments and contact behavior was detected (Cramer’s V = 0.65). Interestingly, as in Bioassay I, the shrimp from the CTL group of Bioassay II avoided contact with the AF after their perception with their antennal flagellum; this was not observed in the other two treatments. Supplementary Video S3 shows a male shrimp displaying the avoidance behavior observed only in shrimp from the CTL group.

Pushing behavior was displayed in a greater number of replicates in the DM treatment (7 replicates), as compared to the VM treatment (5 replicates) and the CTL group (1 replicate) (*p* < 0.05) (Figure 3B). However, a moderate relationship between treatments and pushing behavior was detected (Cramer’s V = 0.37).

Contact and pushing behavior stages occurred more than once in one or more replicates against the AF coated with the DM and VM extracts. Significant differences between the CTL group and the DM and VM treatments were found related to the number of times a contact behavior was displayed per replicate (*p* < 0.01) (Table 3). Similarly, although not statistically significant, the pushing behavior was observed a higher number of times in the DM and VM treatments than in the control group (Table 3).

**Figure 4:**
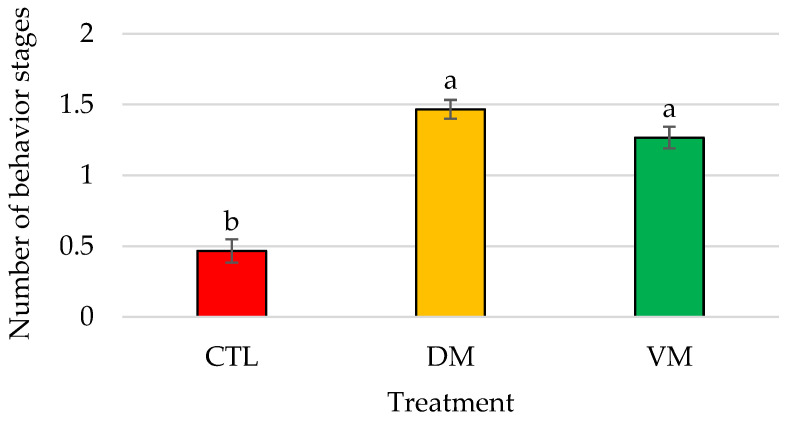
Mean number of different behavior stages recorded expressed as total bioactivity displayed by *Penaeus vannamei* males when exposed to artificial females of Bioassay II (*n* = 15 replicates/treatment); bars represent the standard error of the mean. Different letters indicate significant differences in treatment means (*p* < 0.05). Abbreviations: CTL, control; DM, dorsolateral exoskeleton collected from the abdomen portion of mature female; VM, ventral exoskeleton collected from the abdomen portion of mature female.

Significant differences (*p* < 0.01) in the total bioactivity displayed by males in contact with mature female extracts were found between the CTL group and DM and VM treatments, but not between DM and VM treatments (Figure 4).

## 4. Discussion

Among the behaviors observed, contact and pushing behavior showed a significant association with treatments in Bioassays I and II, and they were recorded more than once in at least one replicate. Both behaviors have been related to reproductive behavior in *P*. *vannamei* by Misamore and Browdy (1996) [15]. These authors described that courtship is initiated by a male push to the female and that direct contact of the antennules of the male with the ventral region of the female is essential to initiate mating. Additionally, contact behavior has been described after sex pheromones exposure in other crustacean species. In this regard, Bauer and Abdalla (2001) reported a higher number of contacts by *Palaemonetes pugio* males with preovulatory females than with immature females [18]. Furthermore, Kelly and Snell (1998) mentioned that in *Trigriopus japonicus*, the number of contacts is dependent on the concentration of contact signals [19]. According to our study, for the extracts collected from immature females, the greatest amount of contact and pushing behaviors was promoted by the extracts collected from the ventral region, whereas, for the extracts collected from mature females, the pushing and contact behaviors seemed to be promoted by the extracts collected from the dorsolateral and ventral region, respectively.

An unexpected observation was the prolonged contact behavior that occurred only in the AF coated with the extract collected from the cuticle of immature females. This behavior lasted longer than 5 min and could be related to the molting stage that the shrimp females were in when they were sacrificed. To ensure that the extracts were obtained from females at stage I of gonadal maturity, sampling was performed once ovarian resorption had concluded, and according to Raviv et al. (2008), in *P*. *vannamei*, ovarian resorption occurs during premolt, one to three days before ecdysis [20]. The existence of a contact signal that attracts *P*. *vannamei* males during the premolt stage of adult females could have two possible antagonistic explanations: (a) induction of cannibalism against molting organisms and (b) stimulation of premolt guarding to protect females. Regarding the first explanation, Wall et al. (2009) demonstrated that in juveniles of the crayfish *Scylla serrata*, the presence of odors during molt does not significantly increase cannibalism compared to odors released when a conspecific is crushed or injured [21]. On the other hand, in juvenile crayfish, *Orconectes rusticus*, a greater tactile response to molting organisms than intermolting organisms have been observed [22]. In these two studies, however, the signals evaluated corresponded to odors, and none of the tactile responses measured lasted more than 10 s. In contrast, in this study, the prolonged contacts lasted more than 30 s and were attributed to contact signals, which could be related to guard behavior, coinciding with Kelly and Snell (1998), who described that mate guarding is a function of contact pheromones [19].

About the possibility of the existence of a premature guard in *P. vannamei*, there were no reports in the literature of the presence of this behavior in this species; in other decapod crustaceans, it is nevertheless common with the objective of protecting the female from other males and possible predators [19,23]. Hardege et al. (2002) mentioned that guarding behavior is a fundamental part of reproductive behavior in crustaceans that breed when the female is in the postmolt stage [24] and, therefore, has been used as a sign of sexual behavior in pheromone identification [25,26]. In *P. vannamei*, however, reproduction occurs when shrimp are in the intermolt period, when both males and females are in the hard-shelled condition [16,20].

The possibility that premature guarding behavior was observed in shrimp males in contact with the AF coated with the cuticular extract from immature females could be based on Parker´s theory, which establishes that in different taxonomic classes of the animal kingdom, courtship behaviors directed to non-receptive females may be exacerbated when the availability of females is low, and may not occur when the availability of females is high [27]. The author explained that when females are scarce, this type of behavior increases the probability of mating so that signals of proximity to receptivity could be adopted to initiate actions designed to monopolize a female prior to mating (e.g., mate guarding). In many *P. vannamei* breeding laboratories, a female:male ratio of 1:0.5–2.8 is maintained, which may explain why this type of behavior has not been observed before [28]. In our bioassays, however, males were kept isolated from females for a prolonged period, which could have induced this behavior, but further research, with choice experiments, testing the effect of extracts collected from mature vs. immature adult females is needed to corroborate this hypothesis.

Concerning total bioactivity, the results obtained from this study also indicate an influence of the treatments on the behavior of males. The fact that both DI and VI treatments of each of the bioassays showed significantly higher bioactivity than the controls, reveals that the signals that promote the different behaviors are distributed throughout the cuticular surface of the females. It is noteworthy, however, that the AF coated with the extracts collected from the ventral region of the females, presented a higher frequency of the higher behavioral stages (pushing and prolonged contact) and a higher average number of repeated behaviors, which could mean that the ventral region has a higher concentration of contact signals in both mature and immature females. Although pushing behavior was more frequently observed in the dorsolateral region when behaviors were evaluated individually, total bioactivity is a more complete response variable, so its interpretation is more accurate.

Notably, none of the treatments promote attachment behavior, which is the unequivocal sign of mating in *P. vannamei* [15]. There are several possible explanations for the absence of this behavior. First, the mature females were sampled over a comprehensive time interval because many females were spawning before sunset. Yano et al. (1988) reported that although mating can occur in the presence of light, the greatest courtship and mating activity occurs between one hour before and one hour after sunset [16]; therefore, it could be that adequate pheromone concentration was not collected given the short period of female attractiveness. Some authors have reported that in crustaceans, the stage of sexual behavior exhibited by males when exposed to potential female sex pheromones is concentration-dependent [5,29].

Another cause could be that males require additional behavioral (movement) or textural stimuli from females to fully exhibit reproductive behavior. In insects and copepods that use contact pheromones, it has been observed that when a receptive female is presented in an immobilized state to males, mating may decrease or never occur [30,31]. Kelly & Snell (1998) proposed that the contact signal also needs a mechanical stimulus to avoid energy expenditure due to the possible interaction with dead females [19]. Furthermore, following the hypothesis of Yano et al. (1988) on the existence of two types of female sex pheromones in *P. vannamei* (contact and distance) [16], it is also possible that the lack of a distance pheromone and that of a mechanical stimulus, in this study prevented the establishment of a complete reproductive behavior.

When analyzing the results obtained from this study, it can be concluded that there is clear evidence that the cuticle of mature and immature females of *P. vannamei* contains chemical messengers that act as contact signals to the opposite sex. The behavioral differences found in the different treatments, however, indicate that there could be two different contact signals with different functions: attraction during premolting and sexual recognition. These different signals could be part of a multimodal communication formed by different components of more than one sensory modality [23]. Therefore, it is possible that a complete reproductive behavior in *P. vannamei* may require the joint action of chemical distance signals, chemical contact signals, and mechanical signals, as hypothesized by other authors [5,16,32]. In other crustaceans, however, the contact signal is sufficient for an attempt of attachment [4].

A trend of higher incidence of contact and higher number of contacts per replicate was observed in males exposed to cuticular extracts from mature females compared to males exposed to cuticular extracts from immature females. The apparent higher incidence of the “no-contact” observation presented by males in the presence of AF coated with extracts collected from immature females, could mean that the putative sexual recognition signal is present in higher concentration in the cuticle of mature females. However, since Bioassay I and II were not performed at the same time, a direct comparison between mature and immature females is not possible due to a possible variation in their experimental conditions, so a bioassay where cuticular extracts from mature females are directly compared with cuticular extracts from immature females is needed.

In Bioassays I and II of the present work, the individuals of the CTL group did not approach the AF after its perception with the antennal flagellum. In contrast, males of the other treatments (DI, VI, DM, and VM) approached to palpate the AF with the antennules after a first perception with the antennal flagellum. This observation is consistent with previous studies that the antennal flagellum may play an important role in contact pheromone perception given the presence of bimodal sensilla [5,32]. Crustaceans have two general types of systems of chemoreception: smell and dispersed chemoreception [11,33,34]. Smell is mediated by sensilla called aesthetascs that are located only in the external or lateral part of the antennules. Aesthetascs are unimodal sensilla innervated by olfactory sensory neurons (OSNs), but not by mechanosensory neurons (MSNs) [33,35]. Dispersed chemoreception, on the other hand, is mediated by bimodal sensilla that are innervated by both chemical sensory neurons (CSNs) and MSNs. Bimodal sensilla are distributed throughout the entire body of the animal and its appendages [36]. This general functioning of chemoreception has also been identified in *P. vannamei* [37]. The perception of pheromones occurs almost universally by “olfactory chemoreceptors”; however, contact pheromones could be captured by dispersed chemoreception [23]. Considering this information, it is hypothesized that, although various organs involved in distance chemoreception can perceive contact pheromones in *P. vannamei*, the antennal flagellum and antennules may play the most important role since they contain the highest number of sensilla [23].

Regarding the possible chemical nature of these potential pheromones, there is a wide variety of molecules used as pheromones in nature, spanning different molecule sizes, structures, functional groups, and combinations, being limited only by the range of molecules that the organism can produce [38]. In the specific case of contact pheromones, they must be relatively insoluble in water (non-polar) to be maintained on the surface of organisms without being constantly replaced [4]. In this work, the contact signals were extracted from the cuticle using hexane as the solvent, denoting the lipid-soluble nature of these signals. Ceramides and hydrocarbons are the primary candidates to act as contact pheromones in *P. vannamei* since they are the liposoluble compounds found in the highest concentration in the crustacean cuticle [39]. Only long-chain cuticular hydrocarbons, however, have been identified as contact pheromones in other crustacean species besides insects [4,40]. Previous studies have posited that hydrocarbons can provide a wide range of information, such as sex, age, physiological state, genetic traits, and mating preferences [41,42]. To confirm the hypothesis that hydrocarbons act as contact pheromones in *P. vannamei*, chemical characterization of the compounds by gas chromatography coupled to a mass spectrometer (GC/MS) is essential.

## 5. Conclusions

In conclusion, the results obtained in this study show that there is a significant effect of liposoluble cuticular compounds from mature and immature females of *P. vannamei* on the behavior of males of the same species, suggesting the presence of contact pheromones, with the extracts from the ventral region generating high bioactivity in shrimp males.

On the other hand, male shrimp exposed to AF with cuticular extracts from immature females displayed three types of behavior: contact, pushing, and prolonged contact, while in the presence of cuticular extracts from mature females, they displayed two types of behavior: contact and pushing. These behaviors suggest that the contact signals found have two functions: attraction during premolting and sexual recognition.

## Figures and Tables

**Table 1:** The scale of activity for the different mating behavioral stages of male *Penaeus vannamei* exposed to artificial females observed in Bioassay I and II.

Degree	Behavior	Description
0	No response	No change in behavior was observed.
1	Contact	The male had at least one contact with the artificial female and initiated an examination with the antennules.
2	Pushing	After contact, the male took impulse to push the artificial female.
3	Prolonged contact	After contact and pushing, the antennules of male maintained constant contact with the artificial female for at least 10 s.

**Table 2:** Mean values ± standard error of the mean number of times a behavior stage was observed in *Penaeus vannamei* males when exposed to artificial females of Bioassay I. Different letters indicate significant differences (*p* < 0.05) between treatments. Abbreviations: (CTL) control; (DI) dorsolateral exoskeleton collected from the abdomen portion of immature female; (VI) ventral exoskeleton collected from the abdomen portion of immature female.

Behavior	Treatment			*p*-Value
	CTL	DI	VI	
Contact	0.40 ± 0.24 b	2.07 ± 0.54 a	2.80 ± 0.50 a	<0.01
Pushing	0.07 ± 0.07 b	0.40 ± 0.16 ab	0.73 ± 0.25 a	<0.05

**Table 3:** Mean values ± standard error of the mean number of times a behavior stage was observed in *Penaeus vannamei* males when exposed to artificial females of Bioassay II. Different letters indicate significant differences (*p* < 0.05) between treatments. Abbreviations: (CTL) control; (DM) dorsolateral exoskeleton collected from the abdomen portion of mature female; (VM) ventral exoskeleton collected from the abdomen portion of mature female.

Behavior	Treatment			*p*-Value
	CTL	DM	VM	
Contact	0.87 ± 0.41 b	2.67 ± 0.51 a	3.60 ± 0.40 a	<0.01
Pushing	0.07 ± 0.07	0.53 ± 0.17	0.40 ± 0.16	0.052

## Data Availability

The original contributions presented in the study are included in the article, further inquiries can be directed to the corresponding author.

## Supplementary Materials

The following supporting information can be downloaded at: https://www.mdpi.com/article/10.3390/ani14111523/s1, Video S1: Contact behavior stage displayed by male shrimp. Evidence of the existence of site-specific female contact pheromones involved in the sexual interaction behavior of the Pacific whiteleg shrimp *Penaeus vannamei*; Video S2: Pushing behavior stage displayed by male shrimp. Evidence of the existence of site-specific female contact pheromones involved in the sexual interaction behavior of the Pacific whiteleg shrimp *Penaeus vannamei*; Video S3: Avoidance behavior displayed by male shrimp. Evidence of the existence of site-specific female contact pheromones involved in the sexual interaction behavior of the Pacific whiteleg shrimp *Penaeus vannamei*.
